# The N-terminal cytoplasmic region of NCBE displays features of an intrinsic disordered structure and represents a novel target for specific drug screening

**DOI:** 10.3389/fphys.2013.00320

**Published:** 2013-11-07

**Authors:** Kaare Bjerregaard-Andersen, Harmonie Perdreau-Dahl, Hanne Guldsten, Jeppe Praetorius, Jan K. Jensen, Jens P. Morth

**Affiliations:** ^1^Norwegian Centre for Molecular Medicine, University of OsloOslo, Norway; ^2^Danish Chinese Centre for Cancer and Proteases, Department for Molecular Biology and Genetics, University of AarhusAarhus, Denmark; ^3^Department of Biomedicine, University of AarhusAarhus, Denmark; ^4^Institute for Experimental Medical Research, Oslo University HospitalOslo, Norway

**Keywords:** SLC4, intrinsic disorder, drug screen, NCBE, IDP, bicarbonate

## Abstract

The sodium dependent bicarbonate transporter NCBE/NBCn2 is predominantly expressed in the central nervous system (CNS). The highest protein concentrations are found in the choroid plexus. The primary function of this integral plasma membrane transport protein is to regulate intracellular neuronal pH and also probably to maintain the pH homeostasis across the blood-cerebrospinal fluid barrier. NCBE is predicted to contain at least 10 transmembrane helices. The N- and C- termini are both cytoplasmic, with a large N-terminal domain (Nt-NCBE) and a relatively small C-terminal domain (Ct-NCBE). The Nt-NCBE is likely to be involved in bicarbonate recognition and transport and contains key areas of regulation involving pH sensing and protein-protein interactions. Intrinsic disordered protein regions (IDPRs) are defined as protein regions having no rigid three-dimensional structure under physiological conditions. They are believed to be involved in signaling networks in which specific, low affinity, protein-protein interactions play an important role. We predict that NCBE and other SoLute Carrier 4 (SLC4) family members have a high level of intrinsic disorder in their cytoplasmic regions. To provide biophysical evidence for the IDPRs predicted in Nt-NCBE, we produced pure (>99%), recombinant Nt-NCBE using *E. coli* as the expression host. The protein was used to perform differential scanning fluorescence spectroscopy (DSF), in order to search for small molecules that would induce secondary or tertiary structure in the IDPRs. We expect this to assist the development of selective pharmaceutical compounds against individual SLC4 family members. We have also determined a low resolution (4 Å) X-ray crystal structure of the N-terminal core domain. The N-terminal cytoplasmic domain (cdb3) of anion exchanger 1 (AE1) shares a similar fold with the N-terminal core domain of NCBE. Crystallization conditions for the full-length N-terminal domain have been sought, but only the core domain yields diffracting crystals.

## Introduction

In general, regulation of intra- and extracellular pH is crucial for cellular function, because most metabolic enzymes have optimal functionality within the narrow pH range of 6.8–7.4. The main buffering system is based on carbonic acid and bicarbonate, which effectively minimizes the impact of short-term pH deviations (Cordat and Casey, 2009). Most mammalian cells depend on selective transport of bicarbonate across the plasma membrane by specific integral membrane proteins belonging to the SoLute Carrier 4 (SLC4) protein family. Ten members of this family are involved in maintaining pH homeostasis (Parker and Boron, 2013). The SLC4 protein family members are divided into three major functional classes; (i) Na^+^-independent Cl^−^/HCO^−^_3_ exchangers, (ii) Na^+^, HCO^−^_3_ co-transporters and, (iii) Na^+^ driven Cl^−^/HCO^−^_3_ exchangers (Boron et al., 2009). The SLC4 family also exhibits differences associated with electrogenic/electroneutral transport characteristics and stilbene derivative sensitivity. Stilbene derivatives, DIDS and SITS, are the classic inhibitors that target the Sodium Bicarbonate Cotransporter (NBC) family. Other potent inhibitors are diBAC oxonol (Liu et al., 2007), S0859 (Ch'en et al., 2008), tenidap and benzamil (Ducoudret et al., 2001). Members of NBC family have earlier been highlighted as potential therapeutic targets of ischemic reperfusion. Inhibition of the electrogenic Na^+^/HCO^−^_3_ cotransporter (NBCe1-b), found in the heart, showed a decrease in ischemic injury (Khandoudi et al., 2001). The family may also be targets to reduce tumor progression in, for example, breast cancer (Boedtkjer et al., 2012). NCBE (NBCn2/SLC4A10) is an electroneutral Na^+^-dependent HCO^−^_3_ transporter (Wang et al., 2000; Giffard et al., 2003; Parker et al., 2008; Damkier et al., 2010) found predominantly in the brain (Liu et al., 2010) with particular high expression levels found in the choroid plexus (Praetorius et al., 2004). NCBE is DIDS-sensitive (Wang et al., 2000). A recent report indicates that NCBE is a potential neuronal drug target. A study performed in mice showed that disruption of the *SLC4A10* gene could prevent fatal epileptic seizures (Jacobs et al., 2008) indicating drugs counteracting this particular NBC might be usable in anticonvulsive therapy. To date no unique inhibitor of NCBE transport activity is known. The development of specific inhibitors against NCBE, or of any SLC4 members in general, would benefit our understanding of the sodium and bicarbonate transport mechanism and perhaps lead to novel drugs that selectively target specific SLC4 family members.

**Figure 1:**
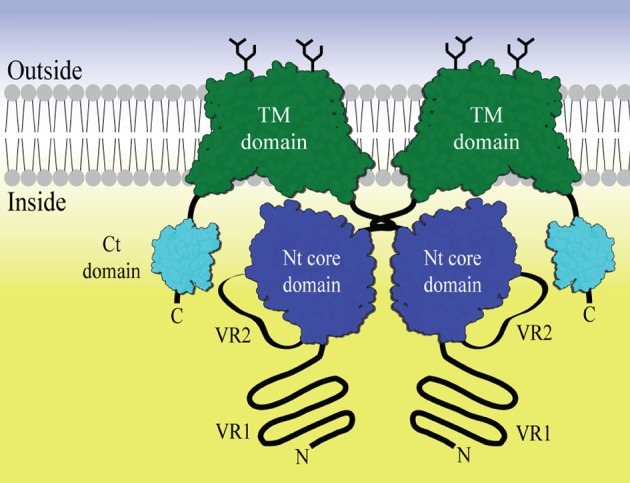
**The general domain structure of a SLC4 bicarbonate transporter represented as a dimer.** The Transmembrane (TM) domain is shown in green. Dimerization is driven by dimerization arms close to the Nt core domains (blue) in the cytoplasm. Variable region 2 (VR2) is drawn as an extended loop from the core domain. Variable region 1 (VR1) is shown as an N- terminal extension from the core domain. The C- terminal region often contains a small PDZ domain (Kennedy, 1995) (cyan). Glycosylations are shown as black sticks.

The general topology of the transporters includes a large amino-terminal (Nt) cytoplasmic domain, a transmembrane domain including extracellular regions of which certain loops can be glycosylated and a smaller carboxy-terminal (Ct) cytoplasmic domain (Figure 1). In general, very little structural data is available for members of this protein family. Two homology models of the anion exchanger 1 (AE1) transmembrane domain based on the *E. coli* chloride-proton channel (ClC) (Bonar et al., 2013) and the uracil transporter (UraA) (Barneaud-Rocca et al., 2013) have been proposed, although there is no significant similarity of AE1 primary structure to these. Historically, the large N-terminal cytoplasmic domain (Nt) has been the target for functional and structural characterization. It is known to be involved in the regulation of transport activity. For example an auto inhibitory domain is found in NBCe1-B/C (McAlear et al., 2006; Lee et al., 2012). Protein—protein and metabolite-protein interactions have also be reported, for example glyceraldehyde-3-phosphate dehydrogenase (GAPDH) with erythrocyte AE1 (Chu and Low, 2006) and inositol-1,4,5-trisphosphate (IP3) receptors binding protein released with IP3 (IRBIT) with NBCe1-B (Shirakabe et al., 2006). However, knowledge of the molecular basis for NBC function and regulation is still limited. The cytoplasmic domain of human AE1 (cdb3) has been crystallized and the structure determined at 2.6 Å resolution. Only residues 55–356 out of the 379 residues available in the crystallized construct were visible in the electron density. The structure forms a dimer, with a dimerization arm formed by residues 317–356. A central elongated β-sheet is present in both monomers surrounded by several α-helices (Zhang et al., 2000). The cytoplasmic Nt domain from NBCe1-A, an electrogenic Na^+^, HCO^−^_3_ co-transporter, has been expressed and purified in *E. coli* (Gill and Boron, 2006), but the structure determination is problematic (Gill et al., 2013). The SLC4 family proteins are, like the ClC channels, believed to be homodimers. Chemical cross-linking followed by SDS-PAGE indicates that membrane bound full-length NBCe1-A forms dimers and traces of tetramers (Kao et al., 2008). AE1 is believed to form both dimers and tetramers (Jiang et al., 2013). Nt-NBCe1-A is able to form monomers, dimers and tetramers in solution (Gill, 2012). It has been observed that the oligomeric state is pH dependent and it is postulated that conformational changes occur within the monomers (Zhang et al., 2000; Gill, 2012). It is therefore possible that the activity of the SLC4 family proteins is related to changes in the oligomeric state and that this happens as a consequence of changes in pH.

Protein function is traditionally thought to depend primarily on the chemical environment created by the tertiary structure of the macromolecule, and it is believed that the composition of the primary structure supports certain secondary and tertiary structures (or folds) of the macromolecule. In recent years, attention has turned to proteins containing non-structured regions—and, in particular, to the function of these regions. These intrinsically disordered protein regions (IDPRs) (Uversky, 2013) are abundant in protein sequences of all organisms, and the number of IDPRs increases as the complexity of the organism increases. In general, the highest percentage of the predicted disorder is found in eukaryotes (Xue et al., 2012) probably because regulation needs to be tighter and is intrinsically more complex than in other domains of life. Experimental identification of IDPRs is tedious and large scale analyses rely on *in silico* predictions from the primary structures. Today disordered regions can be predicted with between 80 and 90% accuracy (He et al., 2009). IDPRs are especially common as mediators of protein–protein interactions. Many are found in proteins related to signaling and are therefore important in understanding diseases such as neurodegenerative disease and cardiovascular disease (Metallo, 2010). For these reasons they represent a class of high-value pharmaceutical targets but due to their poorly characterized properties they have not yet been investigated to any great extent.

In polarized epithelia, the SLC members can reside either in the apical or in the basolateral plasma membranes depending on the primary structure and perhaps the cellular membrane sorting, or membrane protein retaining machineries. To date it is unknown which specific intracellular motifs are involved in the membrane targeting of the SLC4 proteins. Motifs in the Nt or Ct most probably carry a signal for the polarized insertion of the transporters into the plasma membrane (Toye et al., 2004), but extracellular loop glycosylation or even NBC-binding adaptor proteins may also control membrane targeting, as is the case for the Na^+^, K^+^-ATPase (Mellman and Nelson, 2008).

The function of the Nt cytoplasmic domain, in concert with the transmembrane domain, has been studied in AE1 and NBCe1. Unlike AE1 (Groves and Tanner, 1992), NBCe1 seems unable to function without this domain (Espiritu et al., 2006; McAlear et al., 2006). The structure of the link between the cytoplasmic domain and transmembrane domain may therefore be very class specific. Specifically, for NBCe1, a substrate channel formed by the Nt cytoplasmic domain has been suggested (Chang et al., 2008). The Nt cytoplasmic domain, located close to the membrane, consists of two constant regions (CR1 and CR2) separated by variable region 2 (VR2) (Boron et al., 2009). The core domain is N-terminally flanked by variable region 1 [VR1 or Nt-appendage (Parker and Boron, 2013)] and is conserved in all members of the SLC4 family. In this report we have focused on VR1 and VR2 and show that they are likely to be intrinsically disordered in all the SLC4 family members, but do, however, share distinct recognizable signatures. We believe it is this intrinsic disorder that has prevented thorough structural characterization of the cytoplasmic domain. However, we hypothesize that the intrinsically disordered regions found in VR1 and VR2 may be used as specific drug targets to distinguish between the individual SLC4 members and that together they form a distinct structural “fingerprint” for each individual SLC4 transporter. We have identified buffer conditions and a purification protocol to produce pure and monodispersed solutions of both the complete cytoplasmic domain of NCBE and what we refer to as the core domain, without VR1. We also present a novel screening approach, developed to identify small interacting molecules. The screening procedure is potentially applicable as an initial screen to identify leads targeting the VR1 and/or the VR2 regions which are present in the cytoplasmic domain of NCBE and other SLC4 members.

## Materials and methods

### Cloning and expression

The gene encoding *SLC4A10* isoform 3 from *Rattus norvegicus* (rb2NCBE) was purchased from GenScript. Primers were designed for ligation independent cloning (LIC) into the pET-46 Ek/LIC vector (Novagen) for the production of an N-terminal hexa-histidine tagged fusion protein. For construct Nt-NCBE residues 2–421 and the construct Nt-NCBE-ΔD1 residues 96–396. A tobacco etch virus (TEV) protease recognition site was included in the primer located between the hexa-histidine and protein sequence of interest.

Nt-NCBE and Nt-NCBE-ΔD1 were expressed in *E. coli* strain Rosetta2 (Novagen) expressing seven additional tRNAs to compensate for the codon bias in the mammalian gene during prokaryotic expression. An O/N culture of 100 mL LB media with chloramphenicol (50 μg/mL) and ampicillin (34 μg/mL) from a single colony of transformed Rosetta2 cells was made for inoculation of fermentation in 1.5 l of LB media (antibiotics added +1 mL of polyethylene glycol). At optical density measured at 600 nm (OD_600_) of 1.0, the media was cooled to 18°C and induced with 1 mM (IPTG). The cells were harvested after 16 h yielding ~10 g of wet-weight cells. Cell cultures were grown using a LEX HT Bioreactor (Harbinger). Expression levels were visualized by SDS-PAGE with samples taken before and after induction. Protein identity was confirmed using tandem mass-spectrometry.

### Protein purification

For each gram of frozen cell pellet 10 mL lysis buffer (50 mM NaCl, 100 mM Tris-HCl pH 8.0, 1 ug/mL DNAseI, 1 mM PMSF, 1x EDTA-free inhibitor cocktail tablet (Roche), 5 mM β-ME and 40 mM imidazole) was used for resuspension. Resuspended cells were lysed by forcing the suspension three times through high-pressure homogenizer (Emulsiflex C3, Avestin). Cell lysate was kept on ice. The cell lysate was ultracentrifuged for 1 h at 200,000 g to remove cell debris and membrane fractions. The cleared lysate was loaded onto a Ni-NTA column (GE) in equilibration buffer (50 mM NaCl, 100 mM Tris-HCl pH 8.0, 40 mM imidazole and 5 mM β-ME) using an Äkta Prime FPLC system. The column was washed with 10 column volumes (CV) of equilibration buffer. An additional wash with 10 CV of high-salt buffer (500 mM NaCl, 100 mM Tris-HCl pH 8.0, 40 mM imidazole and 5 mM β-ME) was performed. The protein was released with elution buffer (50 mM NaCl, 100 mM Tris-HCl pH 8.0, 200 mM imidazole and 5 mM β-ME). The elution peak fractions were collected and checked by SDS-PAGE analysis. The concentration of the eluted protein was measured using a NanoDrop Spectrophotometer (Thermo Scientific). 1:50 molar ratio of GFP-tagged TEV-protease was added and the suspension was dialyzed O/N against dialysis buffer (50 mM NaCl, 100 mM Tris pH 8.0, 5 mM β-ME and 40 mM imidazole). To remove uncleaved protein and the TEV protease, the suspension was passed over the pre-equilibrated Ni-NTA column and the flow-through containing the cleaved target protein was collected.

The protein was then concentrated using a 10 MWCO Viva spin filter (GE Healthcare). The concentrated sample was loaded on a Superdex 200 HiLoad 10/600 prep-grade column as a final purification step and for determination of homogeneity using an Äkta Purifier HPLC system. Fractions containing pure target protein were collected and kept at 4°C for further analysis. Purity was assessed using SDS-PAGE (Supplementary Figure S1).

### Crystallization and structure determination

Crystallization conditions were screened using commercially available kits. Suitable crystals were only identified for the construct NCBE-Δ D1 in the condition 100 mM potassium thiocyanate (KSCN) and 20% PEG3350. This condition produced showers of crystals of limited size (50 × 50 × 50 μm). Crystals were flash cooled to 100 K in the well solution supplemented with 10% ethylene glycol. Diffraction data was collected at the European Synchrotron Radiation Facility (ESRF) ID29 beamline. Data collection strategy was determined using iMOSFLM (Battye et al., 2011) and the data were processed at the beamline using XDS (Kabsch, 2010) and PHENIX (Adams et al., 2002). A molecular replacement solution was found with the search model for Nt-AE1 [PDB ID 1HYN, (Zhang et al., 2000)]. A homology model of the NCBE-ΔD1 based on 1HYN was created with Modeler (Eswar et al., 2007) and used to perform rigid body refinement against the experimental data. Data and refinement statistics are collected in Supplementary Table S2.

### Differential scanning fluorescence (DSF)

Differential scanning fluorescence (DSF) measurements were carried out using a 7900 HT Fast Real-Time PCR system (Applied Biosystems) using λ_ex_ = 462 nm and λ_em_ = 569 nm (Hawe et al., 2008). The protein stock was 1 mg/mL and contained 100 mM NaCl, 20 mM HEPES pH 7.2, and 0.5 mM TCEP. A prescreening of dye (Sypro Orange, Novagen) and protein was done in order to find the best dye and protein concentration. This was found to be 0.5 mg/mL protein and 100x Sypro Orange. Measurements were carried out in 96 well plate format sealed to prevent evaporation during experiment. Temperature increments of 1°C/min and a temperature range of 20–90°C were used (Niesen et al., 2007). Small molecules were supplied by the Silver Bullets Screen (Hampton Research) and were diluted five-fold for use. Sypro Orange was added as last component. Data treatment was performed using MS Excel and GraphPad Prism 6. A data sheet containing information on individual screen conditions, with hit conditions highlighted, can be found in the Supplementary Material.

### Prediction of disorder in the SLC4 family

Predictions of SLC4 protein disorder were made using the PONDR-FIT metapredictor (Xue et al., 2010). The predictor is the newest development of the PONDR (Predictors Of Natural Disordered Regions) series and shows good accuracy. Query sequences from human SLC4 family proteins were obtained from the UNIPROT sequence database. All isoforms that have been identified at the protein level were chosen for analysis. The average disorder of the whole family, subfamilies and individual isoforms were calculated using GraphPad Prism 6.

## Results

### Crystal structure of N-terminal NCBE core domain

**Figure 2:**
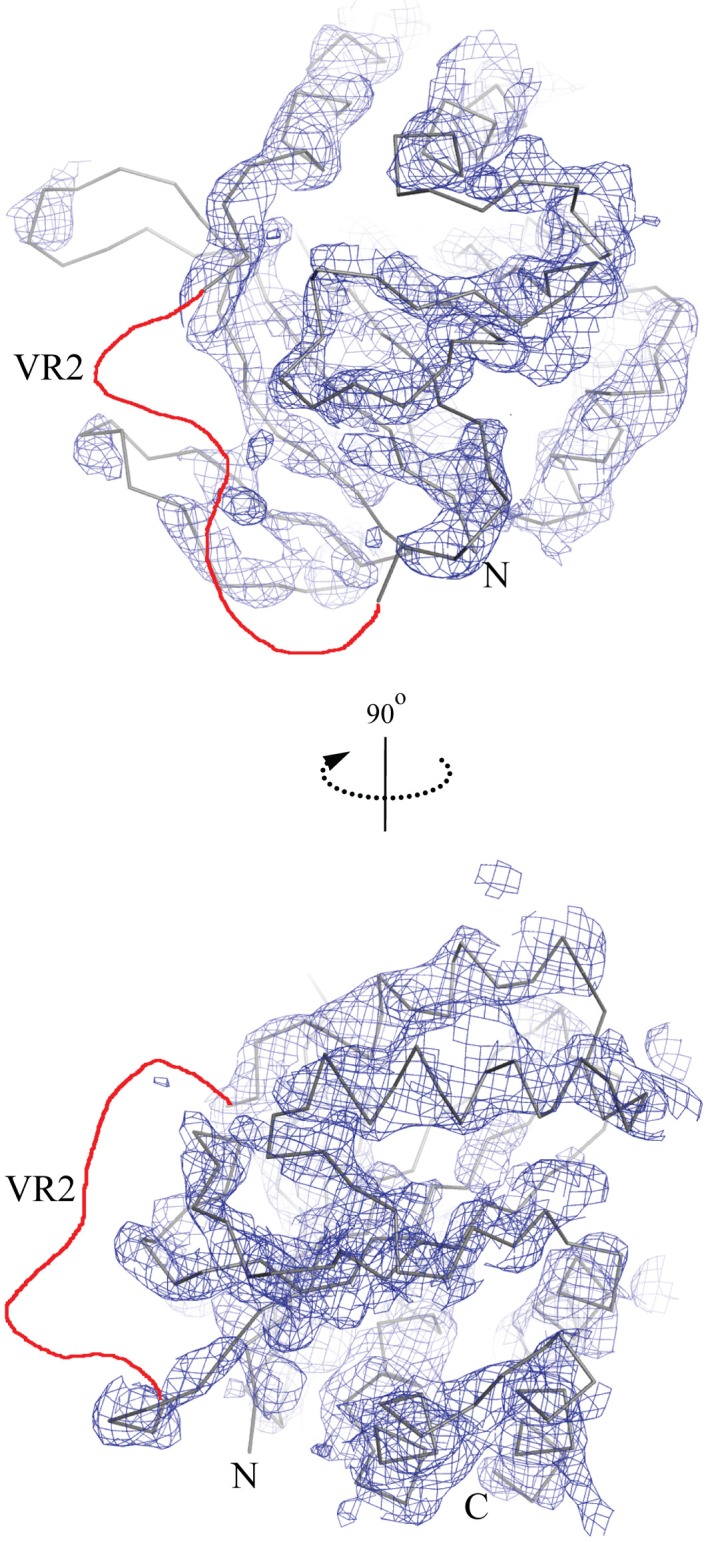
**The low resolution crystal structure of the cytoplasmic N-terminal NCBE core domain.** The lines show a C-α trace of the molecular replacement model structure Nt-AE1 [1HYN, (Zhang et al., 2000)] and the chicken wire shows the corresponding electron density contoured at 1.5σ. Only density for constant regions 1 and 2 (D118-H222 and K293-F389) is visible. No density was observed for variable region 2 (H223-K292) in the structure. The likely position of VR2 is shown as a red line in the figure. Other regions not visible in the electron density were residue ranges 96–117 and 390–396.

A crystallization condition was identified for NCBE-ΔD1, and the crystal structure of the Nt-NCBE core domain was determined at 4.0 Å resolution (Figure 2). At this resolution, ion binding sites and side-chain positions cannot be resolved, only the backbone density is visible for residues 116–222 and 292–389 of the NCBE-ΔD1 construct containing residues 96–396. No electron density is observed for variable region 2 (VR2). There is only one single molecule in the asymmetric unit and no clear dimer with a symmetry related molecule could be found in the crystal lattice. Extensive crystallization screening was undertaken for Nt-NCBE, but no crystallization condition has been identified even after more than 5000 unique conditions have been screened. We suspect that the intrinsic disorder of the Nt-NCBE construct may have prevented crystallization.

### Prediction of disordered regions

**Figure 3:**
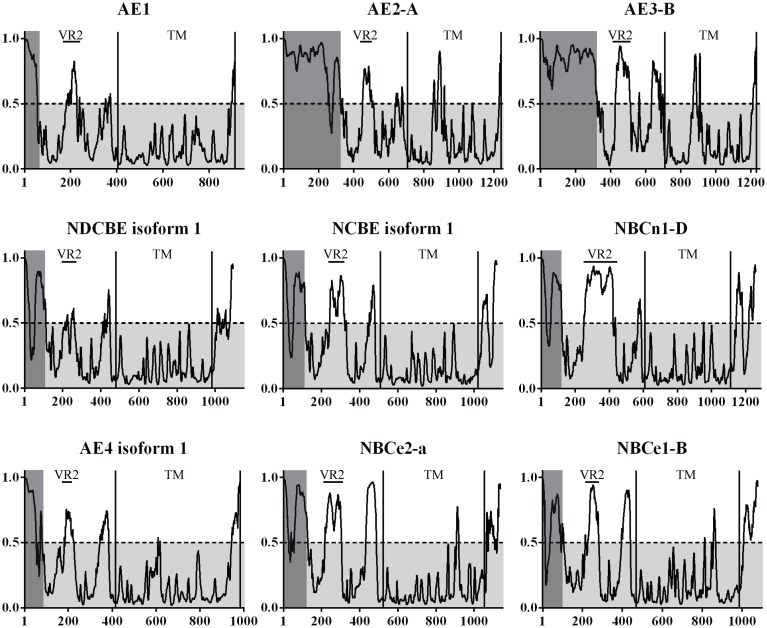
**The prediction of disorder in the bicarbonate transporters of the SLC4 family.** Probability plots of the longest isoforms of SLC4A1-5 and SLC4A7-10 are presented here. A total of 35 isoforms were analyzed. On average, the highest probability of disorder is found in two regions of the N-terminal cytoplasmic domain in the SLC4 family. A high level of disorder is predicted for the variable regions 1 and 2 [VR1, VR2 (Boron et al., 2009)]. VR1 is highlighted in gray. This region was not visible in the Nt-AE1 structure and no experimental structural information exists for it. VR2 is marked on the plots and was modeled in the Nt-AE1 structure. In that structure the high temperature factors and the random coil structure of the region (residues 166–213) support the disorder prediction. VR1 and VR2 are likely involved in protein-protein interactions and regions could constitute a structural “fingerprint” of each individual SLC4 transporter. UniProt accession codes for presented plots are: AE1 (SLC4A 1, P02730), AE2-A (SLC4A 2, P04920-1), AE3-B (SLC4A 3, P48751-1), NDCBE isoform 1 (SLC4A 8, Q2Y0W8-1), NCBE isoform 1 (SLC4A 10, Q6U841-1), NBCn1-D (SLC4A 7, Q9Y6M7-7), AE4 isoform 1 (SLC4A 9, Q96Q91-1), NBCe2-a (SLC4A 5, Q9BY07-1), NBCe1-B (SLC4A 4, Q9Y6R1-1).

A complete and large scale disorder prediction of the SLC4 family was undertaken, as the crystallization trials and partial structure indicated that large disordered regions could be present. All isoforms confirmed at protein level for SLC4A 1-5 and SLC4A 6-11 were analyzed. Selected isoforms are presented in Figure 3. The transmembrane (TM) domain are the most conserved part of the SLC4 family and sequences were aligned to the starting residue of transmembrane helix 1 (TMH1). A high probability of disorder was found for variable region 1 (VR1) of all SLC4 family proteins. Likewise, a rather high probability of disorder was found for variable region 2 [VR2, (Boron et al., 2009)] of the core domain and for the flexible linker region occurring before the TMH1.

### Stabilization of disordered region of extreme N-terminus of NCBE by small molecules

**Figure 4:**
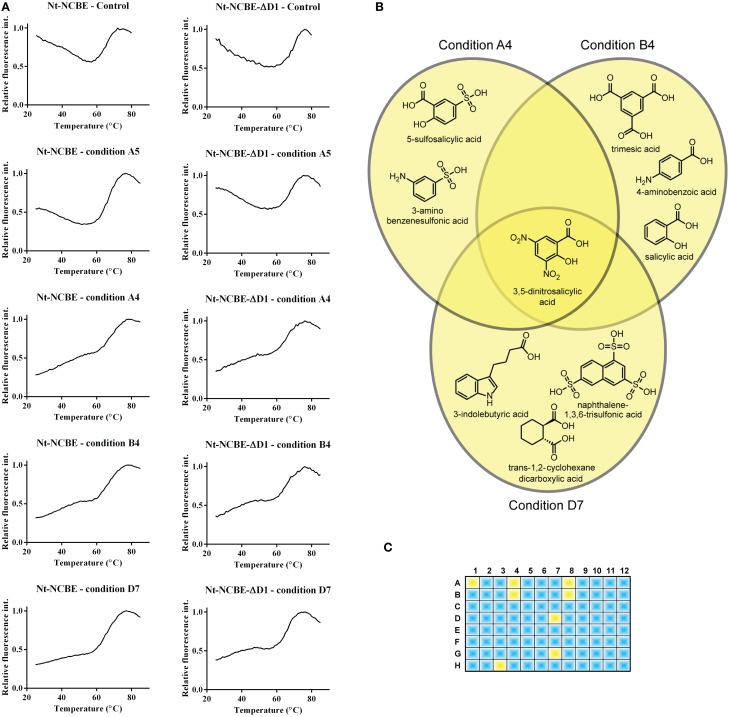
**Screening for interacting small molecules using the thermofluor assay. (A)** Control thermofluor spectra for each construct in the absence of screen molecule showed high initial fluorescence commonly associated with non-folded proteins (Phillips and de la Pena, 2011). Thermofluor spectra of conditions A4, A5, B4, and D7 for both Nt-NCBE and Nt-NCBE-ΔD1 using the Silver Bullets screen: two general profiles were observed. Condition A5 is representative of a “no interaction” profile similar to the control. Conditions A4, B4, and D7 are profiles showing low initial fluorescence and a multi-state melting profile indicating interaction between the construct and the small molecule. We interpret these as weakly stabilized structures. The general hit profiles were found for both Nt-NCBE and Nt-NCBE-ΔD1. The background subtracted relative fluorescence spectra are shown. **(B)** Chemical overlap of the A4, B4, and D7 conditions. All conditions suggest stabilization of the structure by one of the reactive compounds. The conditions have a relatively high chemical similarity, with one compound in common, namely 3,5-dinitrosalicylic acid. We note, however, that a combinatorial effect could be involved in the stabilization of the target protein. **(C)** Hits found in the 96 condition screen. Blue conditions show “no interaction” profiles. Yellow represents “interaction” profiles. 3,5-dinitrosalicylic acid was present in six of eight of these conditions. The identified compounds were not found in any conditions with “no interaction” profiles.

DSF spectroscopy, commonly known as the thermofluor method, is well documented for its use in determining optimal buffer composition and compounds for the stabilization of proteins (Ericsson et al., 2006; Vedadi et al., 2006). Briefly, a hydrophobic molecule (e.g., Sypro Orange) is added to the protein and, optionally, a stabilizing compound. The solution is then heated—as tertiary structure elements reach their melting point, they fall apart and expose their hydrophobic cores which interact with Sypro Orange. The increasing hydrophobic environment enhances the quantum yield of the Sypro Orange chromophore resulting in a dramatic increase of the fluorescent signal. The method is therefore suited for measuring the possible induction of a tertiary structure by small molecules and for high-throughput screening (Pantoliano et al., 2001). Assuming that VR1 and VR2 of NCBE are IDPRs, we would like, if possible, to introduce some tertiary structure to these regions for two reasons. Firstly, to identify compounds that would stabilize a more rigid fold and in turn aid the crystallization process. Secondly, this technique might result in a method for finding leads against pharmacologically interesting IDPRs. The 96 condition Silver Bullets combinatorial library (HR2-096, Hampton Research) provides ~400 unique molecules for this purpose. Overlapping groups of similar compounds are tested in each experiment. In general, a relatively high initial fluorescence was observed for both NCBE constructs in the absence of any screen molecules as well as in the presence of the small molecules in the screen. A high initial fluorescence is often seen for non-globular folded proteins and exposed hydrophobic residues (Phillips and de la Pena, 2011) are anyway expected for IDPRs. The control sample contained 100 mM NaCl and 20 mM HEPES pH 6.8. This melting profile, exemplified by condition A5 in Figure 4A, is considered a non-hit melting profile as we do not see a difference in overall profile from adding small molecules to the protein. A significant change in profile was observed for a number of conditions, for example by conditions A4, B4, and D7 in Figure 4A (third, fourth, and fifth rows). The initial fluorescence starts lower than in the control and the spectra indicate a more complex transition model. We regarded such a profile as indicating an interaction (a hit). Fluorescence backgrounds for the dye and screen molecules alone were subtracted from the spectra. From chemical overlap of conditions we can identify the molecule that is likely to cause the effect (Figure 4B). In total eight hit-profiles were identified from the screen (Figure 4C). Six of the eight hit conditions containing 3,5-dinitrosalicylic acid. Three of the eight hit conditions contained 4-aminobenzoic acid. We, therefore, regard these molecules as candidates for further investigation. The hit molecules were present in concentrations of 0.05% w/v. The remaining spectra and chemical alignments are shown in Supplementary Figure S3.

## Discussion

In general, the predicted levels of structural disorder are high among SLC4 members in comparison to other eukaryotic membrane proteins (Supplementary Figure S4), and therefore we wish to further discuss the IDPRs of that family. The Na^+^-K^+^ ATPase α-1 subunit (Supplementary Figure S4A) and the Cystic Fibrosis Transmembrane conductance Regulator (CFTR) (Supplementary Figure S4B) exhibit disorder profiles that show short spans of disorder (e.g., loop regions) and no extended disordered regions. When analyzing the crystal structure of the Na^+^-K^+^ ATPase α-1 subunit an important loop region can be identified in the disorder profile. The C-terminal of the ClC-channel, however, (Supplementary Figure S4C), exhibits extended disordered regions. This may relate directly to the ClC channel ball-and-chain mechanism of regulation (Grunder et al., 1992). A similar pattern to that of the ClC channel is found for SLC26A6 (Supplementary Figure S4D), a transporter co-occurring with CFTR (He et al., 2010). However, no evidence for a ball-and-chain mechanism for SLC26A6 has been reported. NCBE isoform 1 and NBCn1-D (Figure 3) are shown to illustrate the extent of the disordered regions found in the N-termini of these proteins. The predicted degree of disorder seems to correlate with the fact that many protein–protein interactions have been established for the intracellular domains of SLC4 family proteins. Description of a disordered “fingerprint” formed by VR1 and VR2 of the N-terminal domain could enhance our understanding of the nature of these interactions. We believe that this fingerprint will constitute a unique identifier for each family member. Large variations in the predicted disorder levels are also predicted between isoforms of SLC4 family members (e.g., NBCn1), and it seems that IDPRs may be present at sites of alternative splicing.

IDPRs have previously been thought of as assemblies fluctuating between a 3D structure, maybe statically disordered, and a dynamically disordered state (Tsvetkov et al., 2008). Inhibitors of these fluctuations could be other proteins, metal ions, small molecules and post-translational modifications. The seeding of secondary and tertiary structures can be measured using a range of biophysical methods e.g., circular dichroism (CD), isothermal titration calorimetry (ITC), electron paramagnetic resonance (EPR), and nuclear magnetic resonance (NMR). These are methods applied on a sample to sample basis and are not suited for high-throughput screening (HTS). We show that the thermofluor method provides a high-throughput tool for screening of tertiary structure induction of multiple samples. This method of screening for small molecule interactions with IDPRs could potentially be useful, in conjunction with orthogonal methods, as a first approach to identify pharmacological leads. Our data do not and cannot provide a molecular description of the interaction. However, a lower initial relative fluorescence and a multi-state transition, indicating a stabilization of the structure, provide a selection criterion for HTS methods. Molecules identified by chemical overlap of components of the screen will be candidates for studies using serial methods (e.g., CD or ITC) for further biophysical characterization of the interaction. As with all screening, false positives may occur and many pharmacologically active molecules contain chemical moieties that can absorb light or fluoresce. For this reason we believe it is important to subtract the spectra of the screen molecules and the dye. Fluorescence from screen molecules may interfere with the spectrum if the emission peak is close to that of the used dye. Absorbance may dampen the signal, but we assume this effect is rather small due to the very short light path in the experimental setup and the signal strength usually gained from the dye.

The low-resolution crystal structure represents a first step toward a structural understanding of NCBE. It is also the first crystal structure of a SLC4 protein to be reported since 2006. We show that the fold of the N-terminal core domain is conserved between Nt-NCBE-ΔD1 and Nt-AE1. In AE1 no electron density was observed for residues 1–55 corresponding to VR1 in the crystal structure. VR2 was modeled, but found to be devoid of secondary structure and to have high temperature factors, indicating a large degree of mobility (Zhang et al., 2000) (Supplementary Figure S5). The structure of the fingerprint region remains to be determined, however, the nature of this region as presented in this paper requires methods for studying protein dynamics rather than a crystallographic approach.

Conservation of biological function is often translated directly from sequence conservation at the level of the individual residues, i.e., as changes determined by the chemistry available to the side chain in a defined environment. At the same time the evolutionary selection pressure may work on other levels to conserve protein function. Selection pressure on IDPRs may also work at the biophysical level and to keep a balance between retaining an ability to form a defined structure in the presence of another protein or a metabolite or slipping into a state that cannot be ordered. It could therefore be beneficial to preserve the disorder to prevent order—that is to prevent the formation of secondary structural elements that would hinder the recognition process. We believe there must be such selection pressure to keep the disorder, to prevent the energetically favorable secondary or tertiary structures from forming and thus prevent or induce biological function, and that this is preserved in the fingerprint region. To study this, the experimental approach must be designed to accommodate the specific biophysical characteristics of IDPRs. A common approach to probe protein function is by mutation of key residues—often to alanine to remove amino acid side chain properties. However, in the case of IDPRs one should consider the propensity of alanine to form α-helices when designing a mutational experiment.

The SLC4 family of membrane transporters has proven to be a challenging group of proteins to target for structural and biophysical studies. We hope to elucidate biophysical properties of this group of proteins in order to target individual members selectively with novel inhibitors. The development of these could greatly benefit the field and become valuable tools for further *in vivo* investigations.

## Author contributions

Kaare Bjerregaard-Andersen performed the crystallization experiments and structure determination, performed IDPR predictions, DSF screening, analyzed the data and wrote the paper. Harmonie Perdreau-Dahl and Hanne Guldsten assisted with cloning and purification and analyzed data. Jeppe Praetorius assisted with the data analysis and general physiological comparison. Jan K. Jensen assisted with the biophysical analysis of the DSF data. Jens P. Morth supervised the project, designed experiments and analyzed the data and wrote the paper. All authors commented on the paper.

### Conflict of interest statement

The Authors, Editor and Chief Editor declare that while the author J. Praetorius and the editor E. Bødtkjer are currently employed by the same institution (University of Aarhus, Denmark) there has been no conflict of interest during the review and handling of this manuscript.

## Supplementary meterial

The Supplementary Meterial for this article can be found online at: http://www.frontiersin.org/journal/10.3389/fphys.2013.00320/abstract
