# Supplementary material for: The N-terminal cytoplasmic region of NCBE displays features of an intrinsic disordered structure and represents a novel target for specific drug screening
Source: Front Physiol. 2013 Nov 7;4:320. doi: 10.3389/fphys.2013.00320 (PMC3819638; doi:10.3389/fphys.2013.00320)
Supplement: Supplementary file 1 [file Presentation1.PDF]

Supplementary Material:

S1 – Protein chromatogram and SDS-PAGE

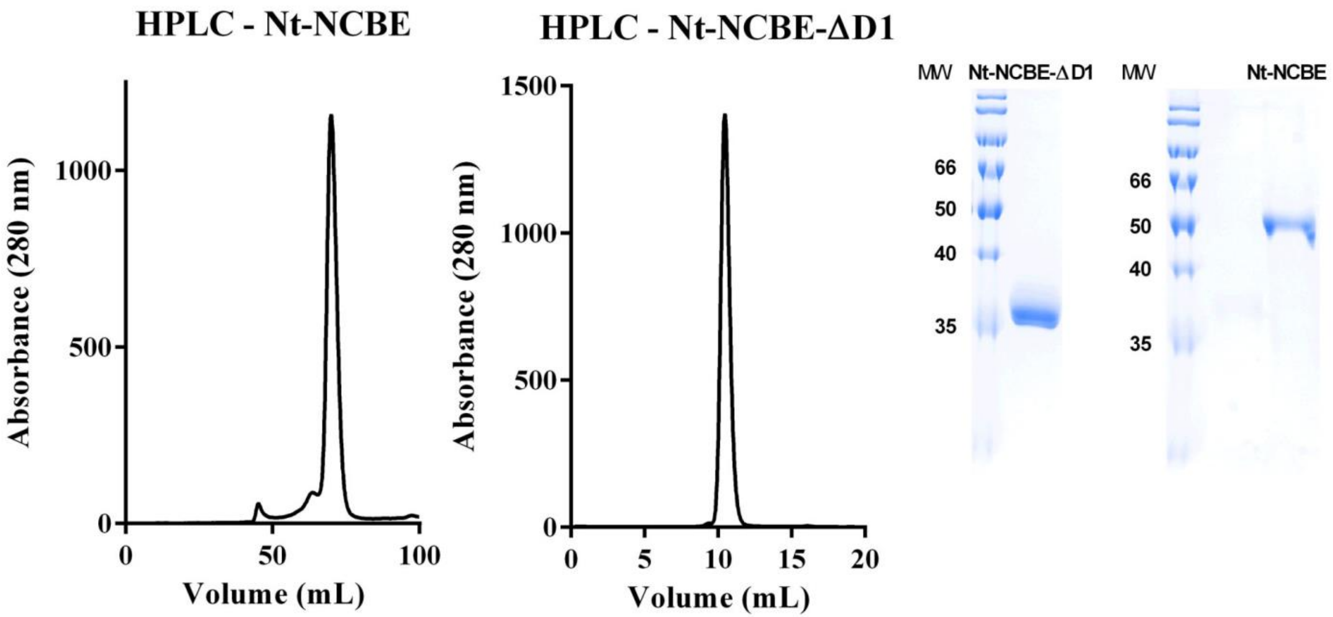

Size exclusion chromatogram and SDS-PAGE. *Nt-NCBE was run on a Hiload Superdex 200 16/600 pg column and Nt-NCBE-ΔD1 was run on Superdex 75 10/300 GL with a buffer composition of 100 mM NaCl, 20 mM Tris-HCl pH 8.0 and 1 mM DTT. The samples show high homogeneity and purity and were subsequently analyzed using SDS-PAGE.*

Supplementary:

S2 - Crystallographic data table:

Table 1. X-ray crystallographic data collection and refinement statistics for NT2-NCBE

	**NT2-NCBE**[1]
**Data collection**	
Beamline	ID-29, ESRF
Wavelength (Å)	1.27
Space group	$C222_1$
Cell dimensions:	
a, *b*, *c* (Å)	82.4, 140.7, 45.8
α, β, γ (°)	90, 90, 90
Resolution (Å)[2]	40.8 - 4.0 (4.1 - 4.0)
R_{merge}[2,3](%)	30.4
$I / \sigma I$[2]	5.0 (2.4)
Completeness (%)[2]	90.8 (93.8)
Redundancy[2]	3.7
Wilson B-factor	95.2
**Refinement**	
Resolution (Å)	4.0
No. reflections[2]	2221 (226)
R_{work} / R_{free}	0.41 / 0.45

[1] Necessary diffraction data was obtained from one crystal

[2] Values in parentheses are for highest-resolution shell.

[3] Redundancy independent R-factor (intensities), (Diederichs, Karplus 1997)

S3 – Thermofluor experiments. The remaining "interaction" conditions from the Silver Bullets screen and the chemical alignment

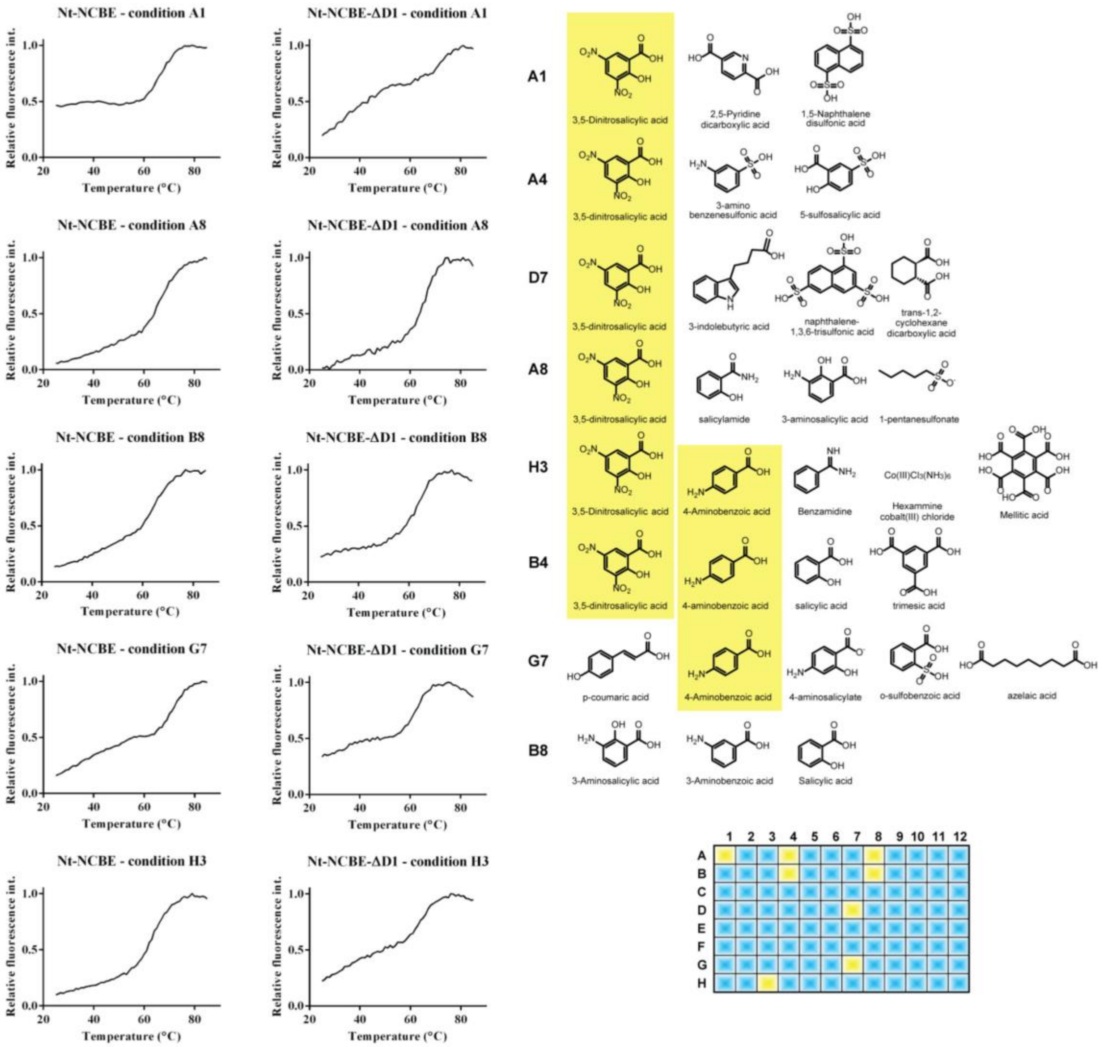

Thermofluor spectra of the remaining "interaction" conditions. *Conditions A1, A8, B8, G7 and H3 all showed a profile with relatively low initial fluorescence. All spectra are background subtracted. A chemical alignment of all hit conditions of the screen is shown on the right. 100% molecular identity overlap is highlighted in yellow. Condition B8 has no chemical identity overlap with other conditions, but a rather a high chemical similarity.*

S4 - Prediction of disorder in the human SLC4 family

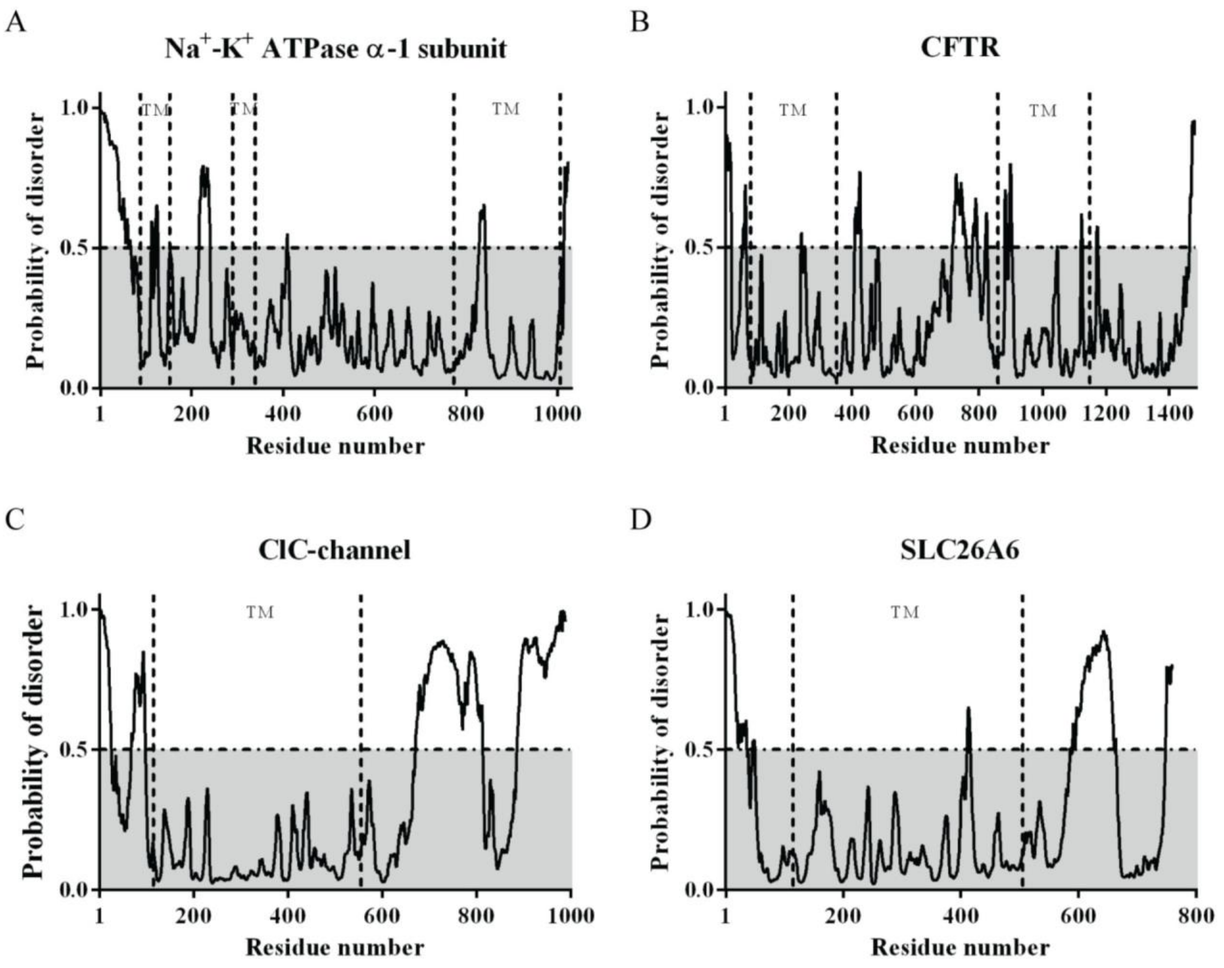

Comparison of disorder profiles from non-SLC4 proteins. *CFTR and Na^{+}-K^{+} ATPase α-1 subunit show patterns of short range disorder which is interpretable as loop regions extending from more rigid structure. SLC26A6 shows similar pattern to the ClC channel with longer stretches of predicted disorder. The ball-and-chain mechanism* (Grunder, Thiemann et al. 1992) *of the ClC channel supports the prediction.*

S5 – Lack of secondary structure elements in the Nt-AE1 variable region

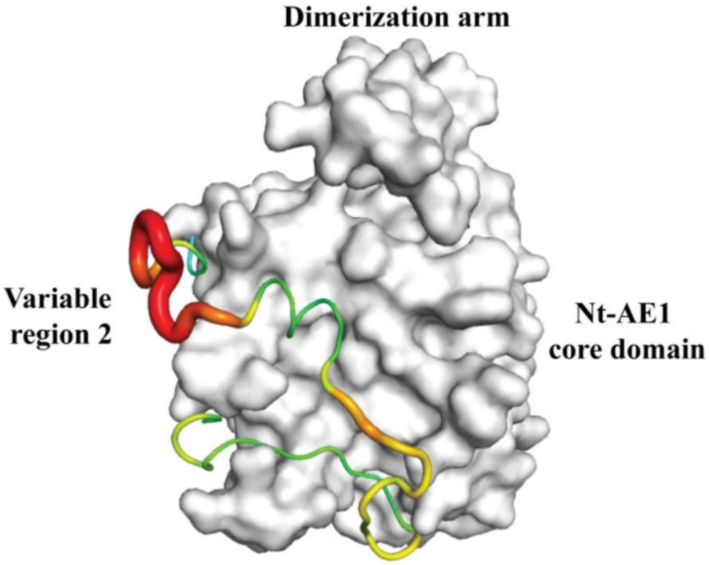

The crystal structure of Nt-AE1 (Zhang, Kiyatkin et al. 2000)**.** *A monomer is shown. The dimerization arm and core domain (CR1 and CR2) are represented as surfaces (white). VR2 is represented as a coil and colored from blue to red according to the temperature-factor (crystallographic B-factor), with red being the highest temperature factors representing the highest mobility. No secondary structure elements are found in the region. The lack of secondary structure elements supports the intrinsic disorder prediction for AE1 (Fig. 3).*
